# Silent Threats to the Liver: Acute Hepatotoxicity Attributed to Selective Androgen Receptor Modulators (SARMs)

**DOI:** 10.7759/cureus.85500

**Published:** 2025-06-07

**Authors:** Bola Habeb, Otto Valdes, Sandy Khair, Erica S Thomson

**Affiliations:** 1 Internal Medicine, University of Florida College of Medicine/Ascension Sacred Heart, Pensacola, USA; 2 Radiation Oncology, Cairo University-National Cancer Institute, Cairo, EGY

**Keywords:** acute hepatotoxicity, acute liver injury, androgen receptors, drug-induced liver injury (dili), muscle growth supplements, non-medical use of sarms, painless jaundice, performance-enhancing drugs, sarms, sarms safety profile

## Abstract

Selective androgen receptor modulators (SARMs) have garnered significant attention in recent years due to their potential to improve muscle mass and athletic performance with reduced androgenic side effects compared to traditional anabolic steroids. However, despite their growing popularity, there is increasing evidence linking SARMs to acute liver injury, raising concerns regarding their safety profile. SARMs can disrupt normal liver function through various pathways, including oxidative stress, alterations in lipid metabolism, and direct hepatocellular toxicity. This article explores the association between SARMs and acute liver injury, examining the mechanisms of hepatotoxicity, clinical manifestations, and potential therapeutic approaches. The findings underscore the need for further research and regulatory oversight in using SARMs, particularly in non-medical and performance-enhancing contexts.

## Introduction

Selective androgen receptor modulators (SARMs) are synthetic compounds that selectively bind to androgen receptors, offering the promise of enhancing muscle mass and bone density while minimizing undesirable androgenic effects such as virilization and prostate enlargement [1]. This selectivity has made SARMs popular among athletes, bodybuilders, and individuals seeking improved physical performance [1,2]. However, despite their favorable pharmacological profile, concerns regarding their safety have emerged, particularly regarding their hepatotoxic potential [2]. The liver, a key organ involved in the metabolism and detoxification of various substances, is susceptible to damage by drugs and chemicals, including SARMs. Drug-induced liver injury (DILI) is a major contributor to acute liver failure (ALF) in the United States, responsible for around 13% of ALF cases [3]. Although SARMs were originally developed for therapeutic applications in conditions such as muscle wasting and osteoporosis, a growing body of case reports and clinical evidence suggests that their use can lead to acute liver injury [1,4]. Recent studies and case reports have documented instances of hepatotoxicity in individuals using SARMs, with symptoms ranging from mild liver enzyme elevations to more severe conditions such as jaundice, cholestasis, and even acute liver failure [4].

The clinical presentation of SARM-induced liver injury typically includes symptoms such as fatigue, jaundice, abdominal pain, and nausea, often accompanied by elevated aspartate aminotransferase (AST), alanine aminotransferase (ALT), bilirubin, and alkaline phosphatase (ALP) [4,5]. In some cases, patients may experience cholestasis or acute liver failure, with more severe manifestations such as hepatic encephalopathy [4,5]. The diagnostic workup begins with a thorough medical history, including the use of SARMs and other medications, followed by laboratory tests to assess liver function, including complete liver panels, coagulation studies, and bilirubin levels. Imaging studies, such as ultrasound, may be used to rule out other causes of liver dysfunction, while a liver biopsy might be considered in severe cases for histological examination. Additionally, ruling out other potential causes of liver injury, such as viral hepatitis or autoimmune liver diseases, is crucial to confirm the diagnosis of SARM-induced hepatotoxicity [3].

The precise mechanisms behind SARM-induced liver injury are not fully understood, but evidence points to various potential pathways, including oxidative stress, mitochondrial dysfunction, and the disruption of normal metabolic processes in hepatocytes [3,5]. Given the increasing use of SARMs in recreational and non-medical settings, understanding the hepatotoxic risks associated with these compounds is crucial for both healthcare providers and users.

Despite these concerns, regulatory agencies such as the FDA have not approved SARMs for non-medical purposes, and their safety remains under scrutiny [1-4]. With increasing reports of acute liver injury, it is imperative to evaluate the implications of SARM use, particularly as it relates to liver health and overall safety. This review aims to consolidate the current understanding of SARM-associated hepatotoxicity and provide insights into the mechanisms, clinical manifestations, and necessary precautions for their use.

## Case presentation

A 27-year-old African American man with no significant past medical history presented with a 10-day history of acute, progressively worsening jaundice and dark-colored urine. He denied abdominal pain, nausea, vomiting, fatigue, or pruritus. The patient reported no history of viral hepatitis, recent travel, sick contacts, tattoos, piercings, or blood transfusions. He denied alcohol use disorder, illicit drug use, and anabolic steroid use; however, he disclosed daily use of RAD-140 (a selective androgen receptor modulator) twice daily for the preceding year. There was no known family history of liver disease.

Clinical findings

Physical examination revealed a temperature of 37.2°C, blood pressure of 122/78 mmHg, pulse of 89 beats per minute (bpm), respiratory rate of 18 breaths per minute, and oxygen saturation of 99% on ambient air. He was alert and oriented to time, place, and person. Scleral icterus was noted on the examination. The abdominal examination revealed a soft, lax abdomen with active bowel sounds. The cardiovascular examination was normal, with a regular rate and rhythm and no audible murmurs. Pulmonary examination demonstrated clear lungs to auscultation with no wheezing, rales, or rhonchi.

Diagnostic assessment

Laboratory results on admission are demonstrated in Table 1.

**Table 1:** Laboratory data obtained on admission. *Abnormal laboratory values. BUN, blood urea nitrogen; AST, aspartate aminotransferase; ALT, alanine aminotransferase; INR, international normalized ratio

Parameters	Patient's values on admission	Reference range, adults
Hemoglobin (g/dL)	14.3	12.0-15.5
Hematocrit (%)	41.2	34.9-44.5
White cell count (per mm^3^)	5,000	3,500-10,500
Platelet count (per mm^3^)	373,000	150,000-450,000
Sodium (mEq/dL)	138	135-145
Potassium (mEq/dL)	4.4	3.5-5.1
Bicarbonate (mEq/dL)	24	22-29
BUN (mg/dL)	12	12-21
Creatinine (mg/dL)	0.9	0.72-1.25
ALT (units/L)	469*	9-29
AST (units/L)	122*	12-31
Alkaline phosphatase (units/L)	131	40-150
Total bilirubin (mg/dL)	9.6*	0.2-1.2
Direct bilirubin (mg/dL)	7.8*	0.1-0.5
Indirect bilirubin (mg/dL)	1.8*	0.0-1.2
INR	1	

Urinalysis was notable for a significant elevation in urobilinogen, though otherwise unremarkable. A comprehensive urine drug screen tested negative for methamphetamine, cocaine, opiates, phencyclidine, methadone, cannabinoids, barbiturates, and benzodiazepines. The patient underwent a comprehensive diagnostic workup to evaluate for infectious, toxic, metabolic, and autoimmune causes, as detailed in Table 2.

**Table 2:** Additional comprehensive diagnostic laboratory workup. Hep A IgM, hepatitis A virus immunoglobulin M; Hep B sAg, hepatitis B virus surface antigen; Hep C Ab, hepatitis C virus antibody; Hep B core IgM, hepatitis B virus core antibody, immunoglobulin M; HIV Ag, Ab, human immunodeficiency virus antigen, antibody; ANA, antinuclear antibody; ASMA, anti-smooth muscle antibody; AMA, antimitochondrial antibody; TIBC, total iron-binding capacity

Parameters	Patient's values	Reference range, adults
	Infectious	
Hep A IgM	Nonreactive	
Hep B sAg	Nonreactive	
Hep C Ab	Nonreactive	
Hep B core IgM	Nonreactive	
HIV Ag, Ab combo screen	Nonreactive	
Herpes simplex virus	Negative	
Epstein-Barr virus	Negative	
Cytomegalovirus	Negative	
Neisseria gonorrhoeae	Negative	
Chlamydia trachomatis	Negative	
	Toxic	
Acetaminophen level (mcg/mL)	<3.0	≤30.0
Salicylate level (mg/dL)	<5.0	5-29
Ethanol (mg/dL)	<10	12-21
	Autoimmune	
ANA	Negative	
ASMA (units)	7	0-19
AMA (U/mL)	1.3	≤3.9
	Metabolic	
Alpha-1 antitrypsin (mg/dL)	124	90-200
Copper (mcg/dL)	93	70-140
Ceruloplasmin (mg/dL)	24	20-35
Iron (mcg/dL)	100	65-175
TIBC (mcg/dL)	320	280-400
Ferritin (ng/mL)	128	21.8-274
Transferrin (mg/dL)	230	174-364

Infectious workup, including serologies for hepatitis A, B, and C; herpes simplex virus; Epstein-Barr virus; and cytomegalovirus, was negative. Screening for human immunodeficiency virus (HIV), *Neisseria gonorrhoeae*, and *Chlamydia trachomatis* also yielded negative results. Evaluation for metabolic liver diseases showed normal alpha-1 antitrypsin, ferritin, copper, and ceruloplasmin levels, excluding alpha-1 antitrypsin deficiency, hemochromatosis, and Wilson's disease. Autoimmune testing revealed normal levels of antinuclear antibody (ANA), anti-smooth muscle antibody (ASMA), and antimitochondrial antibody (AMA), ruling out autoimmune hepatitis. Additionally, serum ethanol, acetaminophen, and salicylate levels were within normal limits, ruling out toxic etiology. Collectively, the diagnostic evaluation did not reveal an infectious, toxic, metabolic, or autoimmune cause of the patient's liver injury.

Imaging studies

**Figure 1:**
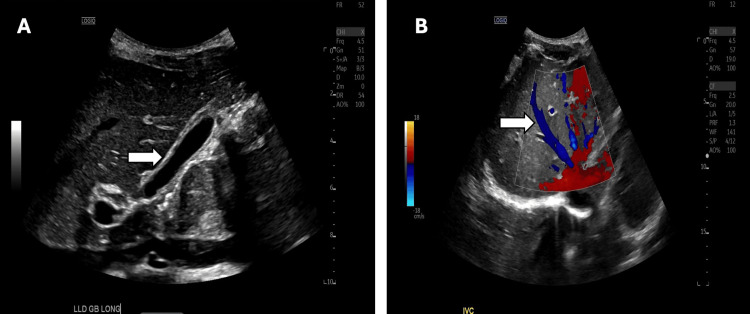
Right upper quadrant ultrasound with Doppler. (A) Contracted gallbladder without evidence of cholelithiasis or biliary ductal dilatation. (B) Normal-caliber portal vein without evidence of portal vein thrombosis.

**Figure 2:**
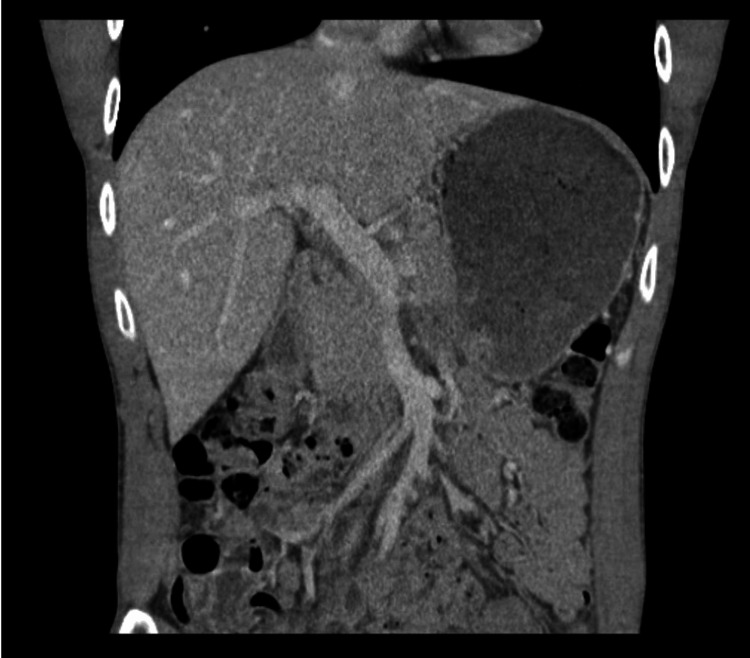
Coronal view of a contrast-enhanced computed tomography (CT) scan of the abdomen and pelvis demonstrating no evidence of acute intra-abdominal pathology.

A right upper quadrant ultrasound with Doppler demonstrated a contracted gallbladder without evidence of cholelithiasis, biliary ductal dilatation, or portal vein thrombosis (Figure 1). Computed tomography (CT) of the abdomen and pelvis with intravenous contrast revealed no acute intra-abdominal pathology, ruling out cancer as the cause of the patient's painless jaundice (Figure 2).

The calculation of the R-factor yielded a value of 9.8, consistent with a hepatocellular pattern of liver injury. Consequently, magnetic resonance cholangiopancreatography (MRCP) was not indicated at that time. With the exclusion of other etiologies, the patient's presentation was considered consistent with drug-induced liver injury secondary to SARMs. Supportive measures were initiated, including intravenous hydration, a clear liquid diet, and anti-emetics. The patient was discharged home on hospital day 3 with recommendations to follow up with gastroenterology in the outpatient setting.

Two weeks later, at his scheduled follow-up, he reported the cessation of SARM use. His laboratory results showed significant improvement in his AST, from 81 to 46 U/L; however, he demonstrated an elevated alkaline phosphatase at 202 U/L and a significant increase in his total bilirubin at 27 mg/dL. The patient was readmitted to the hospital for further evaluation and management. On presentation, he exhibited marked jaundice with scleral icterus and reported significant pruritus. No signs of hepatic encephalopathy were observed, and vital signs were stable. Physical examination did not reveal hepatosplenomegaly or ascites.

**Figure 3:**
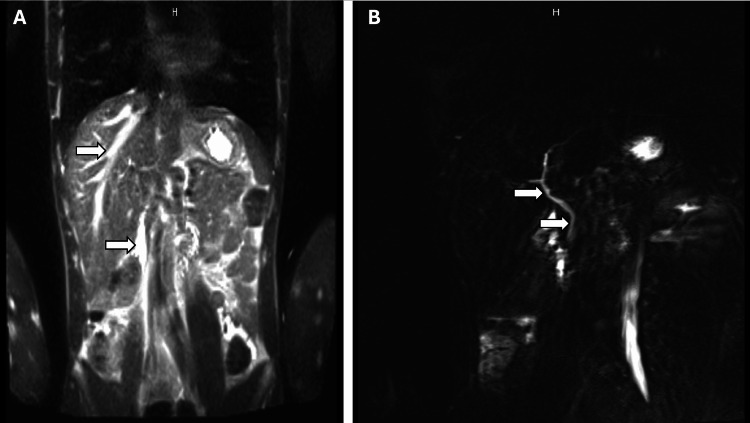
Magnetic resonance cholangiopancreatography (MRCP). (A) Coronal T2-half-Fourier acquisition single-shot turbo spin-echo (HASTE) imaging shows a normal course and caliber of the common bile duct (CBD), without evidence of intrahepatic or extrahepatic biliary ductal dilatation. (B) T2-half-Fourier acquisition single-shot turbo spin-echo (HASTE) with fat saturation (fs), utilizing a slab and three-angle technique, demonstrated a normal course and caliber of the common bile duct (CBD) without evidence of intrahepatic or extrahepatic biliary ductal dilatation.

The calculation of the R-factor on this admission yielded a value of 0.8, consistent with a cholestatic pattern of liver injury. MRCP was obtained, which showed a normal course and caliber of the common bile duct (CBD) with no intrahepatic bile ductal dilatation (Figure 3).

Supportive measures were initiated, including intravenous hydration, anti-emetics, and an antipruritic (cholestyramine). On hospital day 4, the patient was discharged home with a prescription for cholestyramine for pruritus.

**Figure 4:**
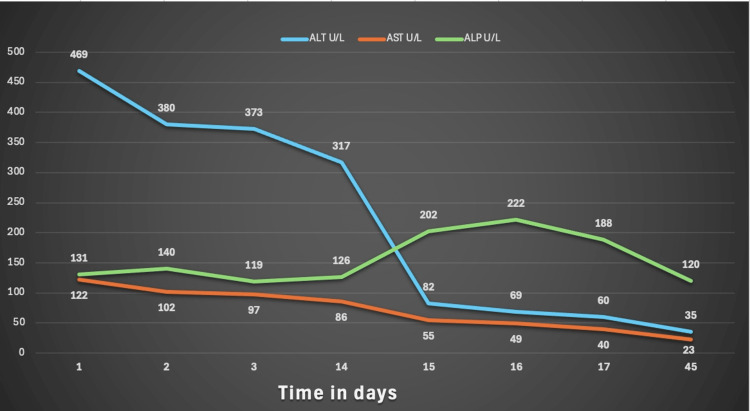
Aminotransferases and alkaline phosphatase trend. Graph showing aminotransferases and ALP trend over 45 days. ALT, alanine aminotransferase; AST, aspartate aminotransferase; ALP, alkaline phosphatase

**Figure 5:**
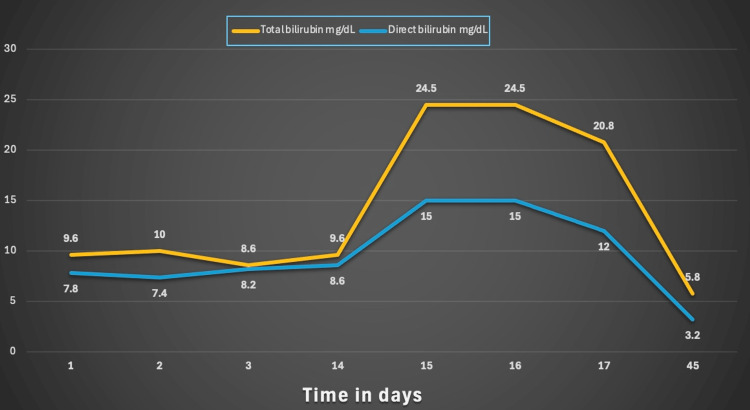
Total and direct bilirubin trend. Notably, total bilirubin peaked at 27 mg/dL and trended down to 5.8 mg/dL at 45 days.

At a one-month outpatient follow-up, the patient demonstrated normalization of liver enzymes and alkaline phosphatase levels (Figure 4). Total bilirubin, which had previously peaked at 27 mg/dL, had decreased to 5.8 mg/dL by day 45 (Figure 5).

Clinically, the patient reported the resolution of symptoms except for mild residual jaundice. He was counseled to engage in moderate physical activity, maintain adequate hydration, and avoid the use of herbal and hormonal supplements.

## Discussion

SARMs have gained popularity in recent years due to their purported benefits in enhancing muscle mass and strength without the significant androgenic side effects commonly associated with anabolic steroids [1]. However, despite their selective mechanism of action, SARMs are not without risks. A growing body of evidence suggests that SARMs can lead to a range of hepatic toxicities, from mild liver enzyme elevations to more severe manifestations such as cholestasis and acute liver failure [3,4].

Though several potential pathways have been proposed, the mechanisms underlying SARM-induced liver injury remain poorly understood. One hypothesis is that SARMs may disrupt hepatic metabolic pathways, leading to oxidative stress and hepatocyte mitochondrial dysfunction [3,5]. These effects can overwhelm the liver's detoxification capacity, resulting in cellular injury and inflammation. Another potential mechanism is the direct modulation of androgen receptors in the liver, which may alter gene expression and enzymatic activity in ways that predispose the liver to injury [3,5]. Furthermore, SARMs may also interfere with bile flow, leading to cholestatic liver injury, as observed in several case reports [3,5].

Clinically, SARM-induced liver injury can present with symptoms such as fatigue, jaundice, right upper quadrant pain, and nausea. Laboratory findings often reveal elevated liver enzymes, particularly ALT and AST, as well as elevated bilirubin levels. Imaging studies may be used to exclude other causes of liver disease, though the definitive diagnosis often relies on the exclusion of other etiologies and a clear history of SARM use [4,5]. In some cases, liver biopsy may be necessary to assess the extent of injury, though it is rarely performed unless the patient is experiencing severe or rapidly progressing liver failure [4,5].

DILI is a well-recognized but uncommon diagnosis that is typically made by excluding other potential causes of liver damage. It is essential to rule out alternative conditions such as viral hepatitis, Wilson's disease (particularly in younger individuals), primary biliary cholangitis, and alcoholic hepatitis. Autoimmune hepatitis should always be high on the differential diagnosis list in suspected DILI cases. Certain medications, including nitrofurantoin and minocycline, are especially known to induce an autoimmune-like form of DILI, often presenting with elevated ANA and ASMA titers [3,6].

Clinically significant DILI is typically defined by any of the following criteria: (1) AST levels exceeding five times the upper limit of normal (ULN) or ALP levels more than twice the ULN, both observed on at least two occasions separated by 24 hours; (2) a total serum bilirubin concentration greater than 42.74 µmol/L accompanied by elevated levels of AST, ALT, or ALP; or (3) an International normalized ratio (INR) above 1.5 in the presence of increased AST, ALT, or ALP levels [3,7]. DILI can result from a wide range of pharmaceutical and herbal agents, with some of the most common culprits including both widely used medications and popular supplements. The spectrum of drugs implicated in DILI is broad, including prescription medications, over-the-counter drugs, and herbal supplements. Common offenders encompass antibiotics such as amoxicillin-clavulanate, nonsteroidal anti-inflammatory drugs (NSAIDs), statins, antiepileptics, and antituberculosis agents [7,8].

Several scoring systems were implemented to determine the likelihood that a specific drug causes liver injury. The R-value, calculated by dividing the ratio of serum ALT to its ULN by the ratio of serum ALP to its ULN, is most accurately determined at the initial presentation. This value aids in classifying the pattern of liver injury: an R-value greater than 5 indicates a hepatocellular pattern, less than 2 suggests a cholestatic pattern, and a value between 2 and 5 reflects a mixed type of liver injury [3]. On initial presentation, the patient had an R-factor of 9.8, indicative of a hepatocellular pattern of liver injury. However, during the second admission, the R-factor decreased to 0.8, consistent with a cholestatic pattern of liver injury. Additionally, the Roussel Uclaf Causality Assessment Method (RUCAM) score is one of the most commonly used tools to assess the likelihood that a drug is responsible for liver injury [6]. It evaluates several factors, including the clinical pattern of liver damage, the time of onset (latency), response to withdrawal of the drug (de-challenge), and the known hepatotoxic potential of the suspected agent. The total score ranges from 0, indicating the drug is unlikely to be the cause, to over 8, which classifies the drug as a "highly probable" cause of liver injury [6]. In our patient's case, a RUCAM score of 7 was calculated during both admissions, supporting a "probable" likelihood that the liver injury was drug-induced.

Despite the clear association between SARMs and acute liver injury in several case reports, the precise prevalence of this complication remains unknown. A review of the current literature identified only a limited number of published cases of SARM-DILI, as summarized in Table 3. All reported cases involved male patients ranging in age from 22 to 52 years. The SARMs implicated included Ligandrol (LGD) and RAD-140. While all patients presented with jaundice, the timing of symptom onset following the discontinuation of the agent varied. None of the individuals had a prior history of liver disease, although three reported episodes of binge drinking on a monthly basis. The peak elevation of liver enzymes differed among cases, and the duration required for liver enzyme normalization ranged from three to 12 months.

**Table 3:** Summary of published case reports on selective androgen receptor modulator (SARM)-induced liver injury. AST, aspartate aminotransferase; ALT, alanine aminotransferase; INR, international normalized ratio; LGD, Ligandrol; NS, not specified; RUQ, right upper quadrant

Author(s)	Age and sex	Clinical presentation	SARM use duration	Alcohol use	AST (U/L)	ALT (U/L)	Bilirubin (mg/dL)	Alkaline phosphatase (U/L)	INR	Platelet count (×10⁹/L)	Management
Mohamed et al., 2023 [3]	22-year-old man	Jaundice, nausea, fatigue, pruritus, dark urine, and light stools for two weeks; scleral icterus observed	RAD-140 (16 weeks)	Monthly binge drinking	118	1,660	Total: 42.75; direct: 29.45	53	0.9	NS	Supportive care; hepatology follow-up
Leung et al., 2022 [5]	24-year-old man	Jaundice, anorexia, nausea, lethargy, and weight loss over five weeks; mild hepatomegaly noted	LGD-4033 (nine weeks)	Monthly binge drinking	111	273	11.6	289	1.0	387	Discontinued SARMs; supportive care
Barbara et al., 2020 [9]	52-year-old man	RUQ pain, jaundice, pruritus, and diarrhea over several weeks; scleral icterus observed	RAD-140 and LGD-4033 (seven weeks)	NS	61	115	6.9	173	1.1	NS	Discontinued SARMs; supportive care with antihistamines
Demangone et al., 2024 [10]	29-year-old man	Jaundice and lethargy over one week; scleral icterus noted	RAD-140 (three months)	Denied alcohol use	73	95	Total: 13.6; direct: 10.4	NS	NS	NS	Discontinued RAD-140, supportive care, and monitoring of liver function tests
Khan et al., 2022 [11]	29-year-old man	Jaundice, fatigue, and elevated liver enzymes were noted after starting SARM supplements	NS	NS	NS	NS	NS	NS	NS	NS	Discontinued SARM supplements; supportive care
Flores et al., 2020 [12]	49-year-old man	Jaundice and pruritus over five weeks; scleral icterus observed	RAD-140 (intermittent for four weeks and four months)	Insignificant	61	115	6.9	173	1.2	347	Discontinued RAD-140, ursodiol and cholestyramine, and hepatology follow-up
This case	23-year-old man	Jaundice, pruritus, dark urine, and elevated liver enzymes and bilirubin	RAD-140 (one year)	Denied alcohol use	122	469	9.6	131	1.0	322	Discontinued RAD-140, supportive care, and ursodiol monitoring of liver function tests

Management primarily involves the immediate discontinuation of the suspected causative agent. Supportive care is essential, addressing symptoms such as pruritus and jaundice. In severe cases, such as those with coagulopathy or hepatic encephalopathy, hospitalization may be necessary [1,3,4]. There is no specific antidote for DILI; thus, monitoring liver function tests and providing supportive care are crucial. Referral to a hepatologist is recommended for cases with progressive liver dysfunction or when liver transplantation is a consideration.​ As SARMs continue to be marketed and used for performance enhancement, it is crucial for healthcare professionals to remain vigilant in diagnosing potential cases of hepatotoxicity and to educate patients about the risks associated with these substances [1,3,4].

## Conclusions

This case report highlights the potential for significant liver injury associated with the use of SARMs, a concern that is increasingly being recognized in both clinical and recreational settings. We emphasize the need for a comprehensive diagnostic workup, including a detailed patient history, liver function tests, and the exclusion of other potential causes of liver injury. Given the increasing misuse of SARMs, it is essential for healthcare professionals to educate patients on the risks associated with these substances, especially considering that their long-term effects remain poorly understood. In light of the potential for severe hepatotoxicity, further research is needed to better understand the mechanisms behind SARM-induced liver injury and to develop more effective strategies for early detection and management.
